# Surfactant protein A reduces TLR4 and inflammatory cytokine mRNA levels in neonatal mouse ileum

**DOI:** 10.1038/s41598-021-82219-y

**Published:** 2021-01-28

**Authors:** Lidan Liu, Chaim Z. Aron, Cullen M. Grable, Adrian Robles, Xiangli Liu, Yuying Liu, Nicole Y. Fatheree, J. Marc Rhoads, Joseph L. Alcorn

**Affiliations:** 1 Department of Anesthesiology, Shengjing Hospital of China Medical University, Shenyang, 110036 China; 2 Division of Neonatal-Perinatal Medicine, Department of Pediatrics, McGovern Medical School, The University of Texas Health Science Center at Houston, 6431 Fannin, Suite 3.222, Houston, TX 77030 USA; 3 McGovern Medical School, The University of Texas Health Science Center at Houston, Houston, TX 77030 USA; 4 Department of Thoracic Surgery, First Hospital of China Medical University, Shenyang, 110001 China; 5 Division of Pediatric Gastroenterology, Department of Pediatrics, McGovern Medical School, The University of Texas Health Science Center at Houston, Houston, TX 77030 USA; 6 Department of Pediatrics, Pediatric Research Center, McGovern Medical School, The University of Texas Health Science Center at Houston, Houston, TX 77030 USA

**Keywords:** Gastrointestinal models, Physiology

## Abstract

Levels of intestinal toll-like receptor 4 (TLR4) impact inflammation in the neonatal gastrointestinal tract. While surfactant protein A (SP-A) is known to regulate TLR4 in the lung, it also reduces intestinal damage, TLR4 and inflammation in an experimental model of necrotizing enterocolitis (NEC) in neonatal rats. We hypothesized that SP-A-deficient (SP-A^−/−^) mice have increased ileal TLR4 and inflammatory cytokine levels compared to wild type mice, impacting intestinal physiology. We found that ileal TLR4 and proinflammatory cytokine levels were significantly higher in infant SP-A^−/−^ mice compared to wild type mice. Gavage of neonatal SP-A^−/−^ mice with purified SP-A reduced ileal TLR4 protein levels. SP-A reduced expression of TLR4 and proinflammatory cytokines in normal human intestinal epithelial cells (FHs74int), suggesting a direct effect. However, incubation of gastrointestinal cell lines with proteasome inhibitors did not abrogate the effect of SP-A on TLR4 protein levels, suggesting that proteasomal degradation is not involved. In a mouse model of experimental NEC, SP-A^−/−^ mice were more susceptible to intestinal stress resembling NEC, while gavage with SP-A significantly decreased ileal damage, TLR4 and proinflammatory cytokine mRNA levels. Our data suggests that SP-A has an extrapulmonary role in the intestinal health of neonatal mice by modulating TLR4 and proinflammatory cytokines mRNA expression in intestinal epithelium.

## Introduction

Despite vast improvements in short-term survival with modern neonatal intensive care, necrotizing enterocolitis (NEC) continues to be one of the most dangerous complications for premature infants, occurring in 10% of extremely low birth weight infants with a death rate over 20%^1^. The presenting signs of NEC are insidious, typically occurring 2–4 weeks after birth, and progression can be rapid with catastrophic results^2^. Since incidence is inversely related to gestation age at birth, prematurity is the greatest risk factor for developing NEC^3^. The precise etiology of NEC is unclear, but evidence points to an excessive inflammatory response resulting from the interaction an immature intestinal epithelium lacking adequate host defense and repair with factors causing stress or injury^4^. The 2006 NICHD workshop on NEC states “NEC can be thought to arise from an uncontrolled exuberant inflammatory response to bacterial colonization that characterizes the intestine of the premature infant”^5^.

The gastrointestinal tract is constantly exposed to exogenous harmful agents as well as to a variety of bacterial species that coexist in the intestinal lumen. To protect itself, the intestinal tract employs both innate and adaptive immune systems as defenses against these insults^6^. At no time is protection of the intestinal mucosa more critical than during birth when the newborn emerges from the relatively protective intrauterine environment to an extrauterine world full of environmental insults. Unfortunately, the defensive mechanisms of the intestinal tract of prematurely-born infants are not fully developed and are functionally inadequate for protection, making the immature intestinal mucosa of neonates more susceptible to bacterial injury and inflammation^7^.

Intestinal epithelium and bacterial pathogens interact through toll-like receptor 4 (TLR4), which recognizes pathogen-associated molecules such as lipopolysaccharides (LPS)^8,9^. These receptors are a crucial part of the innate immune system’s rapid response to pathogens to ward off infection^10^. Upon binding of conserved determinants on microbes, TLRs initiate signaling events to mount an effective immune response and stimulate production of pro-inflammatory cytokines^11^. However, immature neonatal intestine overexpresses inflammatory genes and underexpresses negative feedback regulator genes, making the mucosa prone to exaggerated responses and increasing the risk of inflammatory pathology in the neonatal intestine^7^. For example, NEC is associated with increased expression and activation of intestinal TLR4^12^. Intestinal expression of TLRs and inflammatory cytokines precedes histological injury in experimental animal models of NEC^13^ and TLR4-deficient mice are protected from the development of intestinal damage in these models^14,15^. Injury to the premature intestinal allows inappropriate translocation of bacteria from the lumen into the mucosa and the binding of bacterial LPS to mucosal TLR4, which initiates an innate immune signaling cascade, resulting in increased injury, defective repair, excessive inflammation, apoptosis of enterocytes and intestinal stem cells and inhibition of enterocyte migration and proliferation^16–19^.

While pulmonary surfactant protein A (SP-A) is best known for its roles in preserving the function of pulmonary surfactant to reduce alveolar surface tension^20^, it is also a critical component of the pulmonary innate immune system. SP-A assists in clearance of potential pathogens and has immunomodulatory properties, reducing pro-inflammatory cytokines in the lung^21–23^. SP-A binds LPS and modulates both TLR4 activity^24,25^ and expression in human macrophages and dendritic cells in the lung^24–28^. In the absence of SP-A, alveolar macrophages exhibit increased activation of nuclear factor kappa-light-chain-enhancer of activated B cells (NF-κB)^29^. Long-term exposure of SP-A enhances expression of a negative regulator of TLR4, IL-1 receptor-associated kinase M (IRAK-M), in alveolar macrophages^30,31^, reducing long-term inflammatory responses in alveoli^24,28^. SP-A has the unique ability to reduce short-term inflammation by dampening LPS-induced TLR4 activation as well as reducing long-term excessive inflammation by enhancing of negative regulators of TLR4 activity to signal expression of pro-inflammatory cytokines.

While the lung expresses the highest levels of SP-A, expression is observed in a variety of other tissues including the stomach and intestine^32–34^. We previously reported that orogastric gavage of purified human SP-A to neonatal rat pups significantly reduced intestinal damage, levels of intestinal pro-inflammatory cytokines and levels of intestinal TLR4 levels in a rat pup model of neonatal NEC^35^. While extrapulmonary expression of SP-A has been reported, there are few reports describing an extrapulmonary role for SP-A^32–34^. We hypothesize that SP-A-deficient (SP-A^−/−^) mice have increased ileal TLR4 levels and inflammation compared to wild type mice, impacting intestinal physiology.

## Results

### Ileal TLR4 protein and IL-1β cytokine levels are increased in neonatal and juvenile SP-A-deficient mice compared to wild type mice

**Figure 1:**
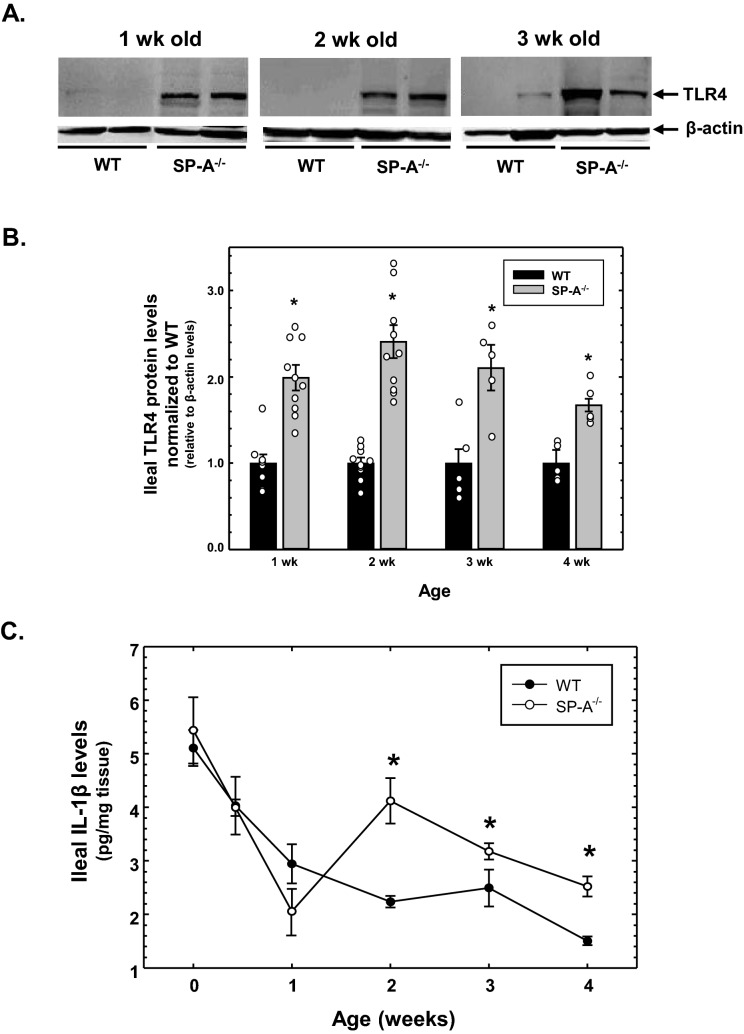
Expression of TLR4 and IL-1β protein in the ileum of SP-A-deficient infant mice compared to wild type infant mice. (**A**) Detection of TLR4 and β-actin protein in the ileum of SP-A-deficient (SP-A^−/−^) and wild type (WT) mouse pups of various ages was accomplished by Western analysis. (**B**) Graphical representation of ileal TLR4 levels in SP-A^−/−^ mice normalized to WT mice at various ages. Shown are the results of WT and SP-A^−/−^ mice for each age (n ≥ 6 from ≥ 2 litters). (**C**) Ileal IL-1β levels, determined by ELISA and expressed as pg per mg ileal protein, in SP-A^−/−^ mice compared to WT as a function of age (n ≥ 6 from ≥ 2 litters). * indicates *P* < 0.05. Figure created using SigmaPlot, ver 14, www.systatsoftware.com.

We previously reported that orogastric gavage with purified SP-A reduced ileal TLR4 and IL-1β levels in neonatal rat pups in an experimental model of NEC^35^. Because wild type rat pups express endogenous SP-A, we wished to determine if ileal TLR4 and IL-1β levels were altered in the absence of SP-A by assessing levels using SP-A^−/−^ mice compared to wild type mice. As seen in representative western analysis in Fig. 1A, TLR4 protein levels were increased in the ileum of 1, 2 and 3 week old SP-A^−/−^ mice compared to similarly aged wild type mice. As shown in Fig. 1B, there was a significant increase (approximately twofold) of ileal TLR4 levels in mice lacking SP-A in all ages tested (1–4 weeks). We then determined if this increase in ileal TLR4 levels was accompanied by an increase in ileal inflammation. As shown in Fig. 1C, ELISA analysis of ileal tissue demonstrated that IL-1β cytokine levels were significantly increased in SP-A-deficient mice from 2 to 4 weeks of age; however, levels of the cytokine were unchanged or significantly lower in younger SP-A^−/−^ mice. It should be noted that mice younger than 2 weeks of age are still nursing and breast milk contains multiple agents that can suppress TLR4-mediated inflammation^36^. While the impact on breast milk on intestinal inflammation in very young neonates is unclear, it is clear that both ileal TLR4 protein and IL-1β cytokines levels are significantly increased in SP-A^−/−^ mice compared to wild type mice.

### Expression of TLR4 mRNA and proinflammatory cytokine mRNA is increased in neonatal SP-A-deficient mice compared to wild type mice

**Figure 2:**
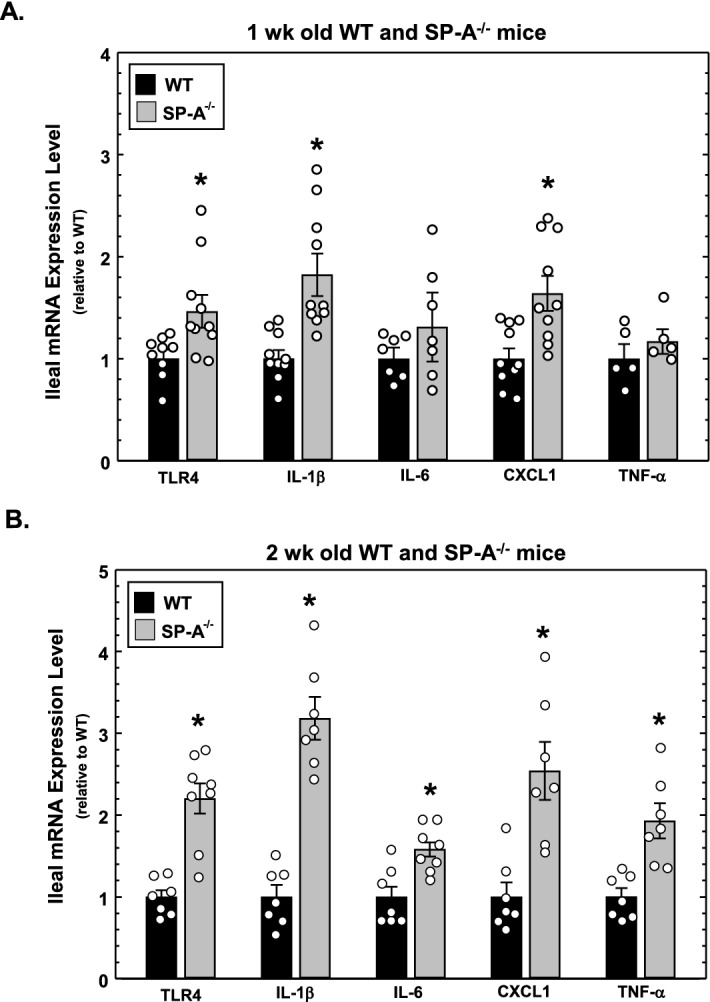
Expression of TLR4 mRNA and proinflammatory cytokine mRNA in the ileum of SP-A-deficient and wild type neonatal mice at 1 and 2 weeks of age. (**A**) Levels of mRNA isolated from the ileum of 1 wk old WT and SP-A^−/−^ mice and assessed by quantitative real-time RT-PCR analysis. Shown are levels of expression relative to WT levels (set as 1). (**B**) Levels of mRNA isolated from the ileum of 2 weeks old WT and SP-A^−/−^ mice and assessed by quantitative real-time RT-PCR analysis. Shown are levels of expression relative to WT levels (set as 1). * indicates P < 0.05, n ≥ 5 from ≥ 2 litters. Figure created using SigmaPlot, ver 14, www.systatsoftware.com.

The results of Fig. 1 indicate that ileal TLR4 and Il-1β protein levels are increased in neonatal and juvenile SP-A^−/−^ mice compared to wild type mice. We next sought to determine if these changes are reflected at the level of mRNA. Using the primers listed in Table 1, we performed real-time quantitative RT-PCR analysis of TLR4, IL-1β, IL-6, CXCL1 and TNF-α mRNA expression in the ileum of SP-A^−/−^ and wild type mice. As shown in Fig. 2A, expression of these mRNAs is increased in 1 week old SP-A^−/−^ mice relative to wild type mice, although only TLR4, IL1-β and CXCL1 expression are significantly increased. On the other hand, expression of all assayed mRNAs is significantly increased in 2 weeks old SP-A^−/−^ mice relative to wild type mice (shown in Fig. 2B) and the difference is greater than that seen in 1 week old mice, reflecting the results of Fig. 1. These results indicate that increases in ileal TLR4 and proinflammatory cytokines occur at the level of mRNA expression in the absence of SP-A.

**Table 1:** Sequence of primers used in real-time quantitative RRT-PCR analysis.

Target	Forward primer	Reverse primer	Amplicon
**Human**			
TLR4	5′-TTTGGACAGTTTCCCACATTGA	5′-AAGCATTCCCACCTTTGTTGG	75 bp
IL-1β	5′-ACAGATGAAGTGCTCCTTCCA	5′-GTCGGAGATTCGTAGCTGGAT	72 bp
IL-6	5′-AATTCGGTACATCCTCGACGG	5′-TTGGAAGGTTCAGGTTGTTTTCT	112 bp
IL-8	5′-ATGACTTCCAAGCTGGCCGTGGCT	5′-TCTCAGCCCTCTTCAAAAACTTCTC	291 bp
**Mouse**			
TLR4	5′-TTTATTCAGAGCCGTTGGTG	5′-CAGAGGATTGTCCTCCCATT	185 bp
IL-1β	5′-GCCACCTTTTGACAGTGATGAG	5′-AAGGTCCACGGGAAAGACAC	218 bp
IL-6	5′-TAGTCCTTCCTACCCCAATTTCC	5′-TTGGTCCTTAGCCACTCCTTC	75 bp
CXCL1	5′-CTGCACCCAAACCGAAGTC	5′-AGCTTCAGGGTCAAGGCAAG	66 bp
TNF-α	5′-CAGCCTCTTCTCATTCCTGC	5′-GGTCTGGGCCATAGAACTGA	132 bp
**Common**			
18S	5′-GTAACCCGTTGAACCCCATT	5′-CCATCCAATCGGTAGTAGCG	150 bp

### Orogastric gavage of SP-A-deficient neonatal mice with purified SP-A reduces ileal TLR4 protein levels

**Figure 3:**
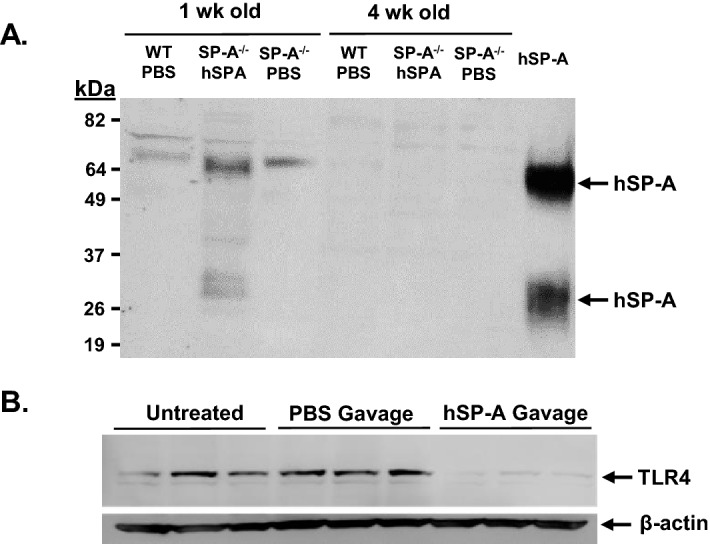
Impact of orogastric gavage of neonatal SP-A^−/−^ mice with purified SP-A. (**A**) Western analysis to detect the presence of SP-A was performed on 1 week and 4 week old WT and SP-A^−/−^ mice 2 h after gavage with 50 µg of purified SP-A. (**B**) 1 week old SP-A^−/−^ mice that were untreated, gavaged with PBS or with 5 µg of purified SP-A in PBS on day 4 and day 6 of life. Shown is Western analysis of TLR4 and β-actin protein in the ileum of 3 mice from each treatment regimen. Figure created using SigmaPlot, ver 14, www.systatsoftware.com.

Because we show that the absence of SP-A results in increased ileal TLR4 and proinflammatory cytokine levels, we then determined if the presence of exogenous SP-A in the ileum could reduce TLR4 protein levels. While our previous investigations demonstrated that its orogastric gavage could alter ileal physiology of neonatal rat pups in an experimental model of NEC, we did not determine the presence of gavaged SP-A in the ileum^37^. Using SP-A-deficient mice to mitigate any interference from endogenous SP-A, we determined if SP-A could be delivered to the ileum via orogastric lavage. One wk and 4 wk old wild type and SP-A^−/−^ mice were orogastrically gavaged with 50 µg purified human SP-A in 100 µl PBS containing trypan blue as a marker. When mice were sacrificed 2 h later, the ileum had been stained with trypan blue indicating transit to that location. Western blot analysis was performed to detect SP-A in the ileum. As seen in Fig. 3A, western analysis demonstrated that SP-A can be detected in the ileum of 1 week old SP-A^−/−^ gavaged with SP-A, but not in similarly aged wild type and SP-A^−/−^ mice receiving only PBS. Interestingly, SP-A was not detectable in 4 week old SP-A^−/−^ gavaged with SP-A, suggesting that while the immature 1 week old stomach could not digest SP-A, the older 4 week old stomach could. These results indicate that orogastric gavage of mice 1 week and younger can deliver SP-A to the ileum. It should be noted that this analysis did not detect the presence of endogenous SP-A protein in wild type mouse ileum.

We then determined if exogenous SP-A could reduce TLR4 protein levels in 1 week old SP-A^−/−^ mouse ileum. Neonatal SP-A^−/−^ mouse pups were gavaged with 5 µg purified human SP-A in PBS or PBS alone at 4 and 6 days of age and sacrificed on day 7 of life. Pups were maintained with dams throughout the experimental period. Western blot analysis shown in Fig. 3B indicates the levels of ileal TLR4 in mice that were either untreated, gavaged with PBS or gavaged with human SP-A in PBS. Orogastric gavage with PBS had little effect on TLR4 levels in the ileum while pups gavaged with SP-A decreased levels of TLR in the ileum.

### SP-A reduces levels of TLR4 and proinflammatory cytokine mRNA in intestinal epithelial cells in cell culture

**Figure 4:**
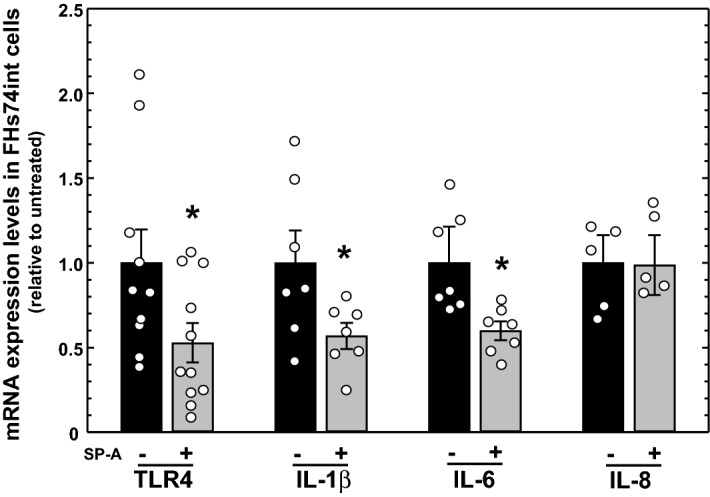
Impact of SP-A exposure on expression of TLR4 and proinflammatory cytokine mRNA in human intestinal epithelial (FHs74int) cells in culture. FHs74int cells were cultured in the absence of presence of SP-A (25 µg/ml) for 24 h. Shown are the results of real-time quantitative RT-PCR analysis of RNA isolated from the cells. All results were normalized to levels determined in untreated cells (set as 1). Shown are the results of 2 determinations, n ≥ 5. * indicates *P* < 0.05. Figure created using SigmaPlot, ver 14, www.systatsoftware.com.

These investigations have demonstrated an extrapulmonary effect of SP-A to reduce TLR4 and proinflammatory cytokine levels in the ileum of the gastrointestinal tract. While it is known that SP-A can directly modulate TLR4 expression and activity in pulmonary alveolar macrophages^24,28^, it is unclear if SP-A can directly modulate TLR4 expression in intestinal epithelial cells. FHs74int cells are non-transformed epithelial cells derived from the small intestine of a normal human fetus that have been maintained in cell culture. FHs74int cells were incubated in the absence or presence of purified human SP-A (25 µg/ml for 24 h) and steady state levels of TLR4 and cytokine mRNA determined by real-time quantitative RT-PCR analysis. As shown in Fig. 4, SP-A significantly reduced TLR4 mRNA relative to cells not exposed to SP-A, suggesting a direct effect of SP-A on TLR4 expression in the intestinal epithelium. In addition, expression of IL-1β and IL-6 mRNA was significantly reduced. While expression of IL-8 mRNA in FHs74int cells was not impacted by the presence of SP-A, we have observed that expression of IL-8 mRNA and protein in FHs74int cells are greater than other cytokines by several orders of magnitude (unpublished results). CXCL1 is mouse homologue of human IL-8, and CXCL1 mRNA levels were impacted by SP-A in mice. Perhaps any effect of SP-A on IL-8 in FHs74int cells is masked by the high levels of IL-8 in these cells. In any case, these results indicate that the reduction of TLR4 and proinflammatory cytokine expression in the ileum by SP-A can be explained as a direct effect of SP-A on the intestinal epithelium.

### SP-A does not reduce TLR4 protein levels in intestinal epithelial cells post-translationally

**Figure 5:**
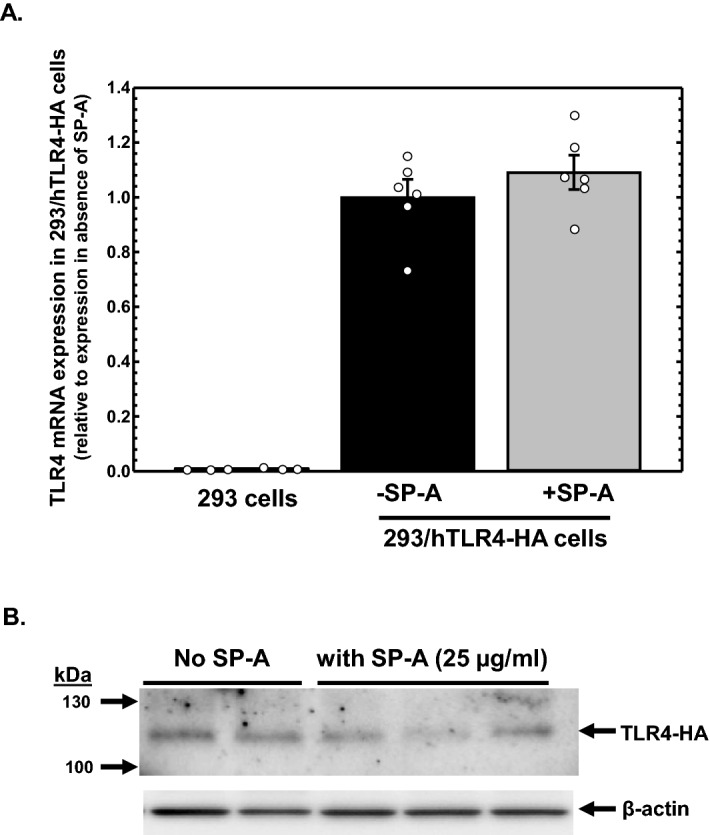
Impact of SP-A exposure on TLR4 mRNA and protein levels in 293/hTLR4-HA cells. (**A**) Real-time quantitative RT-PCR analysis of TLR4 mRNA in HEK293 cells (that do not express TLR4) and in 293/hTLR4-HA cells that express HA-tagged TLR4 via an expression cassette, incubated in the absence or presence of SP-A (25 µg/ml) for 24 h. Shown are the levels of TLR4 mRNA relative to untreated 293/hTLR4-HA cells (n = 6 from 2 assays). (**B**) Western analysis of HA-tagged TLR4protein expressed from 293/hTLR4-HA cells incubated in the absence or presence of SP-A (25 µg/ml) for 24 h. Shown are the results of western analysis using antibodies specific for HA and β-actin following PAGE of cellular protein. Figure created using SigmaPlot, ver 14, www.systatsoftware.com.

We have shown that SP-A can reduce steady-state levels of TLR4 protein and mRNA in the intestinal epithelium, but it is unclear if the mechanism by which SP-A reduces TLR4 protein occurs by impacting TLR4 mRNA levels, protein levels or both. HEK293 cells do not express TLR4^38^, but an HEK293 cell line that has been stably transfected with an expression cassette that expresses hemagglutinin (HA)-tagged TLR4 (293/hTLR4-HA) from the relatively unregulated CMV1 promoter is commercially available. We assessed the ability of SP-A to specifically reduce TLR4 protein levels in 293/hTLR4-HA cells inasmuch as any potential effects of SP-A on transcription or stability of endogenous TLR4 mRNA is absent in these cells. 293/hTLR4-HA cells were incubated in the absence of presence of SP-A (25 µg/ml) for 24 h and the steady-state levels of expressed TLR4 mRNA and protein were determined by real-time quantitative RT-PCR analysis and western analysis, respectively. As can be seen in Fig. 5A, expression of TLR4 mRNA is essentially undetectable in HEK293 cells. TLR4 mRNA expressed from the expression cassette is detectable in 293/hTLR4-HA cells and the presence of SP-A does not alter expression of the TLR4 mRNA. In Fig. 5B, it can be seen that the presence of SP-A does not alter expression of the HA-tagged TLR4 protein compared to untreated cells, suggesting that SP-A does not reduce TLR4 protein levels by activating protein degradation.

**Figure 6:**
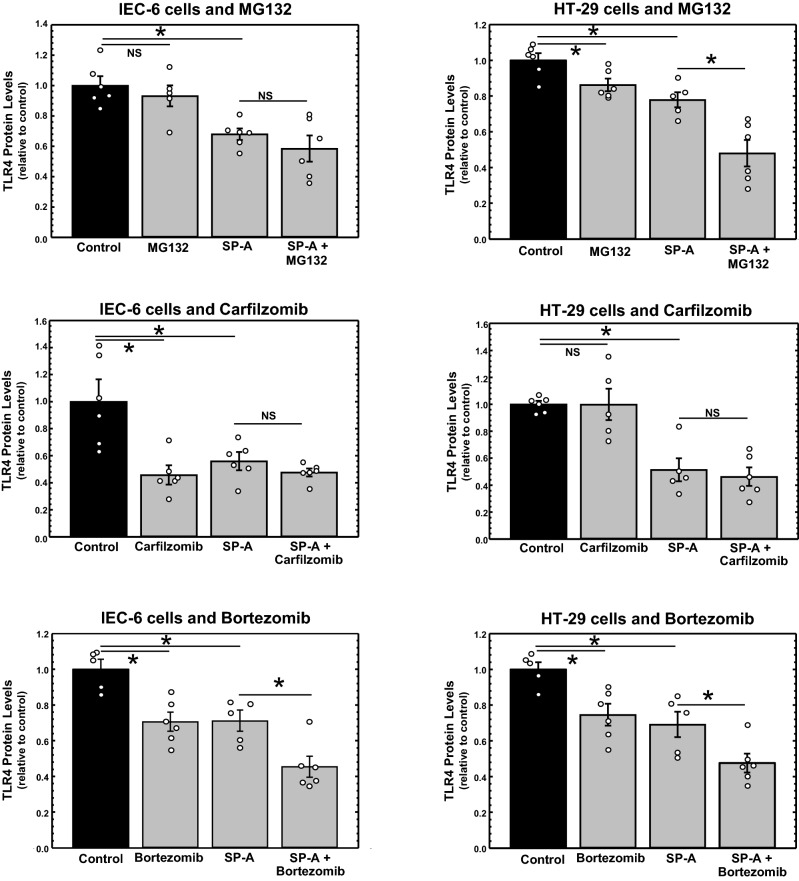
Impact of proteasome inhibitors on TLR4 protein levels in IEC-6 and HT-29 cells incubated in the absence or presence of SP-A. Cells were incubated in the absence or presence of purified human SP-A for 6 h. During the last hour of incubation, MG-132 (20 µM), carfilzomib (20 µM) or bortezomib (20 µM) was added. Shown are the results of 2 determinations, n = 6 from 2 assays. Figure created using SigmaPlot, ver 14, www.systatsoftware.com.

It is known that levels of TLR4 can modulated through ubiquitination by the E3 ubiquitin-protein ligase RNF216, resulting in degradation via the ubiquitin–proteasome pathway^39^. If SP-A reduces TLR4 via proteasomal degradation, we reasoned that treatment of intestinal epithelial cells with proteasome inhibitors would abrogate the ability of SP-A to reduced TLR4 in the cells. HT-29 and IEC-6 ells were cultured in the absence or presence of SP-A (25 µg/ml) for 5 h. Various well-characterized inhibitors of proteasomal activity (MG132, carfilzomib and bortezomib) were added and incubation continued for 1 h until harvest. The levels of TLR4 protein were determined by western analysis. Shown in Fig. 6 are the results plotted as levels of TLR4 protein relative to β-actin protein of each sample normalized to TLR4 levels of untreated cells. As can be seen, the presence of SP-A significantly reduced TLR4 protein levels in all cases, but in no case did the presence of the proteasome inhibitor prevent the inhibitory activity of SP-A on TLR4 levels. These results suggest that while the presence of SP-A reduces steady-state levels of TLR4 mRNA and protein in intestinal epithelial cells, the mechanism of action does not appear to include enhanced proteasomal degradation of TLR4 protein.

### SP-A-deficient neonatal mice are more susceptible to stress-induced intestinal injury than wild type neonatal mice in an experimental model of NEC

**Figure 7:**
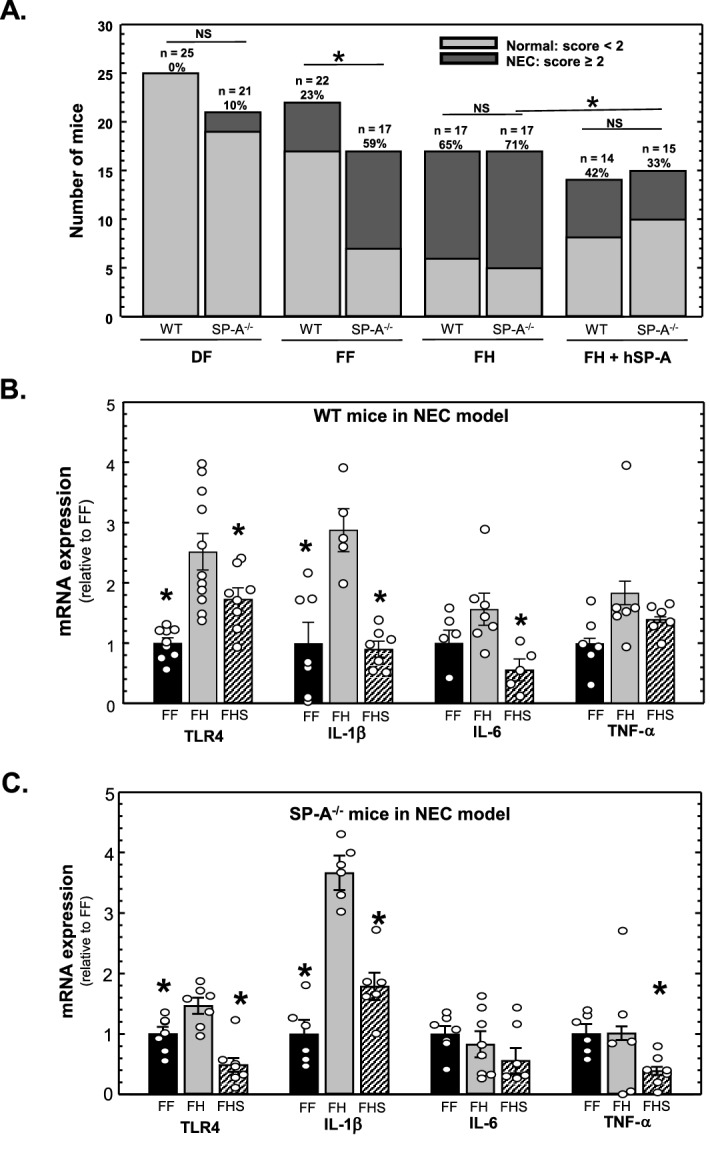
Gavage with SP-A reduces ileal damage, TLR4 and IL-1β in SP-A^−/−^ mice in a neonatal mouse pup model of experimental NEC. (**A**) Assessment of ileal damage corresponding to NEC in WT and SP-A^−/−^ mouse pups. Pups were subjected to the NEC procedure as described in “Methods”. *FF *formula fed, *FH *formula-fed + hypoxia, *FHS *formula-fed + hypoxia + gavaged human SP-A. Shown are the numbers of pups with scores suggesting little damage (assessment < 2) or NEC-like damage (assessment ≥ 2) in each group. * indicates P < 0.05, from ≥ 3 litters. (**B**) Expression of TLR4 mRNA and proinflammatory cytokine mRNA isolated from the ileum of WT mice subjected to the NEC procedure and assessed by quantitative real-time RT-PCR analysis. Shown are levels of expression relative to FF levels (set as 1). * indicates P < 0.05, n ≥ 6 from ≥ 2 litters. (**C**) Expression of TLR4 mRNA and proinflammatory cytokine mRNA isolated from the ileum of SP-A^−/−^ mice subjected to the NEC procedure and assessed by quantitative real-time RT-PCR analysis. Shown are levels of expression relative to FF levels (set as 1). * indicates P < 0.05, n ≥ 6 from ≥ 2 litters. Figure created using SigmaPlot, ver 14, www.systatsoftware.com.

This study has shown that levels of TLR4 protein and IL-1β inflammatory cytokines are increased in the ileum of SP-A-deficient mice, suggesting that the intestine of SP-A-deficient mice may be more susceptible to risk of inflammatory pathology in the neonatal intestine. We used an established model of experimental NEC involving neonatal mouse pups (8–10 days old)^40^ that requires exposure to a variety of stresses (formula feeding, hypoxia, hypothermia) to determine the susceptibility of SP-A-deficient mice to intestinal stress. Wild type and SP-A^−/−^ mice were divided into 4 groups; (1) dam-fed, (2) formula-fed, (3) formula-fed with hypoxia and hypothermia and (4) formula-fed with hypoxia and hypothermia with daily gavage of purified human SP-A (5 µg) to deliver SP-A to the intestine as shown in Fig. 3. Formula-feeding alone is a known risk factor for NEC in premature neonates and episodic hypoxia and hypothermia is meant to reproduce gastrointestinal reperfusion-like damage^1–3^. After the course of exposure in the model was completed, tissues were harvested for histochemical analysis. The incidence and severity of intestinal damage resembling NEC in the sections was determined by a scoring system (0–4) developed for use in neonatal rodents where the severity of damage was graded by three separate masked evaluators, with histological scores of ≥ 2 defined as having NEC^41^. As shown in Fig. 7A, there was no significant difference in NEC-like intestinal damage comparing dam-fed wild type and SP-A^−/−^ neonatal pups. However, under the stress of formula feeding alone, 59% of SP-A^−/−^ neonatal pups had damage resembling NEC, which was significantly higher than the wild type neonatal pups (23%). Upon exposure to the combined stresses of formula feeding, hypoxia and hypothermia, the amount of damage resembling NEC was high in both wild type (65%) and SP-A^−/−^ (71%) neonatal pups, but there was no significant difference. As shown in Fig. 7A, and as expected from previous investigations using neonatal rat pups, orogastric gavage of purified SP-A (5 µg/day) to SP-A-deficient pups treated with all stressors was sufficient to significantly reduce damage resembling NEC (p = 0.036).

We then assessed the impact of orogastric gavage of purified SP-A on ileal proinflammatory cytokine expression in this model of experimental NEC. In Fig. 7B are shown the results of real-time quantitative RT-PCR analysis of ileal TLR4 and proinflammatory cytokine mRNA expression in neonatal wild type mice. As can be seen, formula feeding with hypoxia + hypothermia (FH) significantly increased TLR4 and IL-1β mRNA levels compared to formula feeding (FF) alone. Administration of purified SP-A (FHS) significantly reduced expression of TLR4, IL-1β and IL-6-compared to the FH group. Similar results are seen in Fig. 7C which shows that expression of ileal TLR4, IL-1β, and TNF-α mRNA in neonatal SP-A^-/-^ mice subjected to experimental NEC is significantly reduced upon orogastric gavage with purified SP-A.

## Discussion

Traditionally, SP-A activity is thought to be limited to the lung, but numerous reports of extra-pulmonary SP-A expression^42^ suggest that the function of this highly conserved component of the innate immune system may be important beyond the lung. SPA-deficient (SP-A^−/−^) mice have no obvious phenotype other than enhanced susceptibility to pulmonary infections^43,44^. However, it was reported that neonatal and juvenile SP-A^−/−^ mice housed in a non-hygienic environment have significant mortality that was associated with significant gastrointestinal tract pathology rather than the expected lung pathology^45^. Furthermore, that study showed that gavage of SP-A^−/−^ pups with purified human SP-A improved newborn survival. We previously reported that orogastric gavage of purified human SP-A to neonatal rat pups significantly reduced intestinal damage and levels of intestinal TLR4 levels in a rat pup model of neonatal NEC^35^. Here, we furthered those investigations using mice deficient in the SP-A gene. We found that intestinal levels of TLR4 and inflammatory cytokines are increased in SP-A^−/−^ mice compared to wild type mice. In addition, the presence of exogenous SP-A via orogastric gavage reduces ileal TLR4 protein levels. SP-A does not appear to increase degradation of TLR4 through the proteasomal pathway. We also show that neonatal SP-A^−/−^ mice are more susceptible to stress that leads to intestinal damage resembling NEC and that exposure to exogenous SP-A can reduce ileal damage, TLR4 levels and inflammatory cytokine levels. SP-A is known to modulate TLR4 activity and inflammation in the lung and we now report a novel activity of SP-A to modulate TLR4 levels and inflammation in neonatal mouse intestine.

While the results described here indicate that SP-A has a role in modulating TLR4 and inflammatory cytokine expression in the ileum, the source of SP-A and mechanism of action is still unclear. The results using cell lines suggest that SP-A can have a direct effect to reduce TLR4 and cytokine mRNA expression in the ileal epithelium, but the impact of gavaged SP-A in neonatal mice could be direct, indirect or both. Gastrointestinal expression of SP-A has been reported ^32–34^, but the evidence is not very strong. We find that expression of SP-A mRNA in the ileum of adult (8 week old) mice is approximately 0.6% of that expressed in lung tissue, and expression of SP-A in the ileum of neonatal mice is barely detectable by real-time quantitative RT-PCR with threshold cycles in the range of those observed when ileal RNA isolated from SP-A-deficient mice is used (unpublished results). In these investigations we found that endogenous mouse SP-A was not detected in 1 week and 4 week old mouse pups by western analysis (Fig. 3A). Another complicating factor in these studies is the fact that SP-A has been reported to influence the immunomodulatory properties of breast milk^46,47^ as well as impacting levels of IgA found in breast milk^48^. Realizing that the pups used in these investigations are being nursed, the complexities of the interaction of breast milk with inflammatory character of the gastrointestinal tract are not addressed here and could be contributing to the unexpected findings shown in Fig. 1C. These results can only definitely state that increased TLR4 and proinflammatory cytokine expression in the ileum occurs in the complete absence of SP-A expression throughout the body.

SP-A has been reported to suppress pro-inflammatory cytokine production in alveolar macrophages through upregulation of IRAK-M expression^24^, a negative regulator of TLR-mediated NF-κB activation of cytokine transcription^31^, which suppresses the TLR4 signaling cascade. However, SP-A did not alter TLR4 expression in alveolar macrophages in those studies. SP-A has been shown to directly interact with TLR4 to reduce inflammation^49^, and a 20 amino acid-long fragment of SP-A has been shown to reduce inflammation in colonic epithelial SW480 cells^50^. We have observed that TLR4 levels protein are increased in lung alveolar epithelial cells of SP-A^−/−^ mice compared to wild type mice, and TLR4 expression is markedly reduced in human alveolar epithelial A549 cells cultured in the presence of SP-A compared to cells cultured in the absence of SP-A (unpublished data). We suggest that SP-A reduces TLR4-mediated inflammatory responses in epithelial cells by reducing levels of TLR4 mRNA, a mechanism that is distinctly different from the ability of SP-A to reduce TLR4-mediated inflammatory responses in macrophages by suppressing the signal cascade initiated by activation of TLR4.

NEC is responsible for ~ 7% of all neonatal intensive care unit admissions, exacting huge healthcare costs (up to $1 billion annually)^1^. As complications occur before diagnosis of NEC and treatment is primarily supportive, surgical removal of affected bowel is frequent. While prematurity is the greatest risk factor for developing NEC^3^, the rate of feeding and type of feeding (formula > breast milk) is also associated with NEC. In two studies, 90% of infants with NEC were fed with infant formula (instead of breast milk)^51,52^. Hypoxia and damage to the intestinal mucosa are also considered risk factors, as NEC is thought to result from a reperfusion injury that stimulates an inflammatory cascade^51,52^. The presence of abnormal microbial flora along with additional risk factors of bowel ischemia/perforation, immaturity of host mucosal defense and enteral feedings is thought to contribute to the initiation and pathogenesis of NEC, likely due to an excessive inflammatory response^13,53,54^. Although the precise mechanisms responsible for initiation of NEC remain unclear, intestinal inflammation is key in its pathophysiology. While the innate immune system is essential in defense of pathogenic bacteria in the intestine, when over-stimulated or left unchecked, it may also contribute to inflammatory injury. Immature host defenses in premature intestine may lead excessive inflammation, consistent with the finding that human fetal intestinal epithelial cells produce higher levels of inflammatory cytokines in response to bacteria compared with adult intestinal epithelial cells^7^.

In large part, TLR4-mediated inflammation plays an important role in inflammatory diseases in the intestine^55^. One of the first histopathologic events in experimental models of NEC is caused by TLR4 activation in immature small intestinal tissue, but not in adult tissues^56,57^. Feeding with cow-based infant formula in both humans and animals is associated with increased activation of intestinal TLR4, and expression of TLRs and intestinal inflammatory cytokines precede observable injury in animal models of NEC^13^. Moreover, physiological stressors associated with the development of NEC (LPS and hypoxia) sensitize the intestinal epithelium through up-regulation of TLR4^12^. Taken together, these studies suggest that interactions of LPS from bacteria and TLR4, resulting in excess proinflammatory cytokines in premature intestinal epithelium, lead to intestinal damage and increase susceptibility of the neonate to intestinal diseases. A critical role TLR4 in these processes was demonstrated in TLR4 mutant mice which have reduced intestinal TNF-α levels resulting from formula-feeding and stress compared to wild type mice^58^, and in TLR4 null transgenic mice which are protected from the development of intestinal damage in experimental NEC models^14,15^.

Protection of the relatively immature intestine of the neonate comes from a variety of sources, such as epidermal growth factor (EGF) found in amniotic fluid (AF). SP-A can also provide protection to the immature intestine of the neonate, but it is unclear as to how sufficient levels of SP-A are delivered to the intestine. Expression of SP-A in the lungs is developmentally regulated; SP-A is detected in the lungs at 19 wks gestation in humans and expression increases dramatically after 32 wks gestation^59,60^, but very little is known concerning the ontogeny of SP-A in the intestine. Importantly for this study, the levels of SP-A in AF in humans has been shown to increase from < 3 µg/ml at 30 weeks gestation to > 24 µg/ml at term^60–63^. The changes in amniotic SP-A levels mirror that in the fetal lung, suggesting that SP-A in AF is from the lungs where secreted SP-A is transmitted to the AF through in utero fetal breathing movements. Thus, the most plausible source of SP-A for the intestine is the lung, and a mechanism to deliver SP-A from the lung to the gastrointestinal tract during fetal development is through AF. The fetus at term swallows up to a liter of AF (and SP-A) per day^61^, providing a rich source of SP-A to the immature intestinal mucosa at term. Interestingly, it has been reported that AF reduces inflammation and TLR4 activity in premature mouse intestine through the action of EGF, but in that study, the gestational age of the AF was unclear, as was the presence of SP-A^46^. We propose a model in which the presence of SP-A reduces TLR4 levels and inflammation in neonatal intestine, which lessens the possibility of intestinal inflammation when exposed to environmental insults (Fig. 7). SP-A is secreted from the developing fetal lung late in gestation and accumulates in AF. AF is swallowed by the fetus, providing a rich source of SP-A throughout the gastrointestinal tissue at term that reduces intestinal levels of TLR4 and inflammation. Prematurity is the greatest risk factor for the development of NEC in infants. Because expression of SP-A in the lungs is not initiated until the third trimester of gestation, premature delivery of infants (< 32 weeks gestation) increases the immature nature of neonatal intestine and will interrupt the natural in utero delivery of SP-A to the intestine, resulting in a situation where the immature intestine is predisposed to excessive inflammation and damage when exposed to the extrauterine environment, facilitating the development of intestinal diseases such as NEC. While this theory is not addressed by the findings here, we do show that SP-A does have a role in immunomodulation of the gastrointestinal tract.

Given that current technology cannot diagnose NEC in its early stages and that there are no treatments for the condition, development of prophylactic/treatment strategies to reduce the inflammatory environment in the premature GI tract is a worthy research goal. We have shown that orogastric gavage can deliver SP-A to neonatal intestine resulting in blunting of intestinal inflammation. Understanding the physiological role of SP-A in modulating excessive inflammation in neonatal intestine and the molecular mechanism by which SP-A reduces TLR4 in epithelial cells can potentially lead to the use of SP-A as a biomarker for and prophylactic agent against the onset of excessive inflammation that can lead to intestinal diseases such as NEC in premature infants.

## Methods

This study was carried out in compliance with the ARRIVE guidelines.

### Animals

Mice used in this study were housed in the Center for Laboratory Animal Medicine and Care (CLAMC) at the McGovern Medical School, Houston, TX. Wild type C57BL/6J mice (WT) were purchased from The Jackson Laboratory (#000664, Bar Harbor, ME). Mice deficient for the SP-A gene (SP-A^−/−^) were obtained from Dr. Carole Mendelson^64,65^, and were originally generated in the lab of Dr. Samuel Hawgood, who backcrossed the SP-A^−/−^ locus to a C57B/6 background^66^. To reduce genetic and intestinal microbiome differences in our wild type and SP-A^−/−^ lines used in the investigations, we crossed our SP-A^−/−^ line with wild type C57BL/6 mice purchased from the Jackson Laboratory and the resulting heterozygotes from different breeding pairs were backcrossed to regenerate the lines. We repeat the backcross process on a yearly basis and house all lines in the location in our animal facility so they receive the same food, bedding and water. Studies were approved by the Animal Welfare Committee of the University of Texas Health Science Center at House (HSC-AWC-17-028) and thus, all methods were carried out in accordance with relevant guidelines and regulations.

### NEC procedure

Induction of NEC in mouse pups was performed using the technique developed by Liu et al.^40^ in which formula feeding combined with exposure to stress (hypoxia and hypothermia) induces NEC, but is not associated with excessive early mortality. Seven to 10-day old mouse pups were separated from dams, housed in an incubator and starved for 12 h before initiation of orogastric formula feeding with 200 µl formula via a Nutriline polyurethane neonatal PICC catheter (#1252.31G; Vygon, Lansdale, PA), four times daily for 4 days. The formula consisted of 15 g Similac 60/40 (Ross Pediatrics, Columbus, OH) in 75 ml of Esbilac canine milk replacement (Pet-Ag, Hampshire, IL) and contains 1.86 kcal/ml, which meets the calculated calorie intake required for maintenance of energy in newborn mice (∼ 200 kcal·per kg per·day). Stress-induced NEC was mediated through hypoxic exposure (5% O_2_–95% N_2_ for 10 min in a hypoxia chamber [Billups-Rothenberg, Del Mar, CA)] followed by hypothermia (4 °C for 5 min) twice daily for 4 days. Animals were monitored every 3 h during the 4-day study period. No analgesia was necessary for mice in this study or in previous publications by other groups^56,58,67,68^. Pups were euthanized on day 5 of feeding for tissue collection. If pups exhibited pain, demonstrated labored respirations, exhibited abdominal distension, or had gastrointestinal bleeding, they were euthanized.

### Tissue harvest and NEC evaluation

Following incision of the abdomen, the gastrointestinal tract was carefully removed. The small intestine was evaluated visually for typical gross signs of NEC, such as intestinal distension, wall hemorrhage, or necrosis. The last 5 cm of terminal ileum was excised. Parts of ileum for each animal were placed immediately in liquid nitrogen for RNA (2 cm) and protein (2 cm) isolation. The terminal 1 cm of ileum was fixed in 10% formalin, processed by the Cellular and Molecular Morphology Core Laboratory (the Texas Medical Center Digestive Disease Center, Houston, TX) and stained with hematoxylin and eosin for histological evaluation. Pathological changes in intestinal architecture were quantified using a modified NEC scoring system^67,69,70^. Histological changes in the ileum were scored by 3 blinded evaluators on a scale of 0 (normal), 1 (mild, separation of the villous core, without other abnormalities), 2 (moderate, villous core separation, submucosal edema, and epithelial sloughing), and 3 (severe, denudation of epithelium with loss of villi, full-thickness necrosis). Animals with histological scores 2 were defined as having NEC.

### Cell lines

All experimental protocols involving the use of cell lines were approved by the Environmental Health and Safety Department of UTHealth. The human colonic HT-29 epithelial cell line (#HTB-38; ATCC, Manassas, VA), the rat small intestine IEC-6 epithelial cell line (#CRL-1592; ATCC, Manassas, VA) human fetal FHs74int epithelial cells (#CCL-241; ATCC, Manassas, VA), the HEK293 cell line (#CRL-1573; ATCC, Manassas, VA) and the 293/hTLR4-HA cell line (#293-htlr4ha; InvivoGen, San Diego, CA) were used in this study. HT-29 and IEC-6 cells were cultured in Dulbecco's Modified Eagle's Medium (DMEM) supplemented with 10% fetal bovine serum (FBS; #12303C; Sigma-Aldrich, St. Louis, MO) and antibiotic/antimycotic (1%, #15240062; ThermoFisher Scientific, Waltham, MA). 293/hTLR4-HA cells were incubated in DMEM with 10% FBS with 50 U/ml penicillin, 50 µg/ml streptomycin, 100 µg/ml normocin. FHs74int cells were cultured in Hybri-Care Medium (#46-X; ATCC, Manassas, VA) supplemented with 30 ng/ml epidermal growth factor (hEGF; #E9644, Sigma-Aldrich, St. Louis, MO), 10% FBS and penicillin/streptomycin (1%). All cells were maintained in a humidified 5% CO_2_ incubator.

### Purification of human SP-A

SP–A was purified as previously described^71,72^ from human bronchoalveolar lavage fluid (BAL) collected from alveolar proteinosis patients and generously provided by Dr. Francis X. McCormack at the University of Cincinnati. Purity was assessed by silver stain of PAGE separated SP-A. Endotoxin content of the purified SP-A (0.26 pg/µg SP-A) was determined with a ToxinSensor Chromogenic LAL Endotoxin Assay Kit (# L00350; Genscript, Piscataway, NJ), and the protein stored at − 80 °C.

### Western analysis

Cells and ileum were homogenized in RIPA buffer (#SC-24948; Santa Cruz Biotechnology, Inc., Santa Cruz, CA) supplemented with Halt protease inhibitors (#78430; ThermoFisher Scientific, Waltham, MA). Homogenates were subjected to western blot analysis as described previously^13^. Antibodies specific for SP-A (#sc-13977; Santa Cruz Biotechnology, Santa Cruz, CA), TLR4 (#IMG-5031A; Imgenex Corp, San Diego, CA), hemagglutinin HA-tag (#RT0268; Bio X Cell, Lebanon, NH) and β-actin (#ab6276, Abcam, Cambridge, MA) were used to detected the proteins after PAGE. Complexes were visualized using the ECL Plus Western Blotting Detection System (#RPN2135, Amersham Biosciences Corp, Piscataway, NJ) and quantified on a Storm 840 Phosphorimager (GE Healthcare, Piscataway, NJ). Original images of western analysis shown in Figs. 1A, 3A and 5A are provided in Supplementary Information.

### Real-time quantitative RT-PCR of mRNA

Tissue or cells were treated with Trizol (#15596026, ThermoFisher Scientific, Waltham, MA), as per the manufacturer’s instructions, and homogenized at full speed using a rotor–stator homogenizer. mRNA was then isolated, treated with Turbo DNA-free™ kit (#100789371; ThermoFisher Scientific, Waltham, MA), and purified using a RNeasy^®^ mini kit (#160040219; Qiagen, Germantown, MD) according to the manufacturer’s instructions. cDNA was synthesized from of total RNA (1 µg) using an iScript cDNA Synthesis Kit (#170-8891; Bio-Rad Laboratories, Hercules, CA). For qPCR analysis, primers listed in Table 1 were used. qPCR was performed using a LightCycler 480II System (Roche Diagnostics, Indianapolis, IN) and the iTaq Universal SYBR Green Supermix (#1725124; Bio-Rad Laboratories, Hercules, CA) as per the instructions of the manufacturers. Data were calculated by the comparative C_T_ method (C_T_, threshold cycle)^73,74^. Data are plotted as fold increases in amplicon CT values normalized 18S RNA.

### Cytokine measurement

Tissue was homogenized in RIPA buffer containing proteinase inhibitors and levels of IL-1β in the homogenate were determined by ELISA as per the manufacturer’s instructions (#MLB00C; R&D Systems, Inc, Minneapolis, MN). Levels are expressed as pg per mg tissue.

### Statistical analysis

Animal and cell data represent means ± SE with a vertical scatter plot of the individual data used to generate these values. Statistical analysis of protein and mRNA levels was performed using the student’s t-test with Sigmaplot software, version 12. Statistical analysis comparing assessment of NEC between the groups was performed by Chi-square analysis. A value of *P* < 0.05 determined by either method was considered significant.

## Supplementary Information

Supplementary Information.

## Data Availability

No datasets were generated or analyzed during the current study.
