# Supplementary material for: Surfactant protein A reduces TLR4 and inflammatory cytokine mRNA levels in neonatal mouse ileum
Source: Sci Rep. 2021 Jan 28;11:2593. doi: 10.1038/s41598-021-82219-y (PMC7843620; doi:10.1038/s41598-021-82219-y)
Supplement: Supplementary file 1 — Supplementary Information. [file 41598_2021_82219_MOESM1_ESM.pptx]

## Slide 1
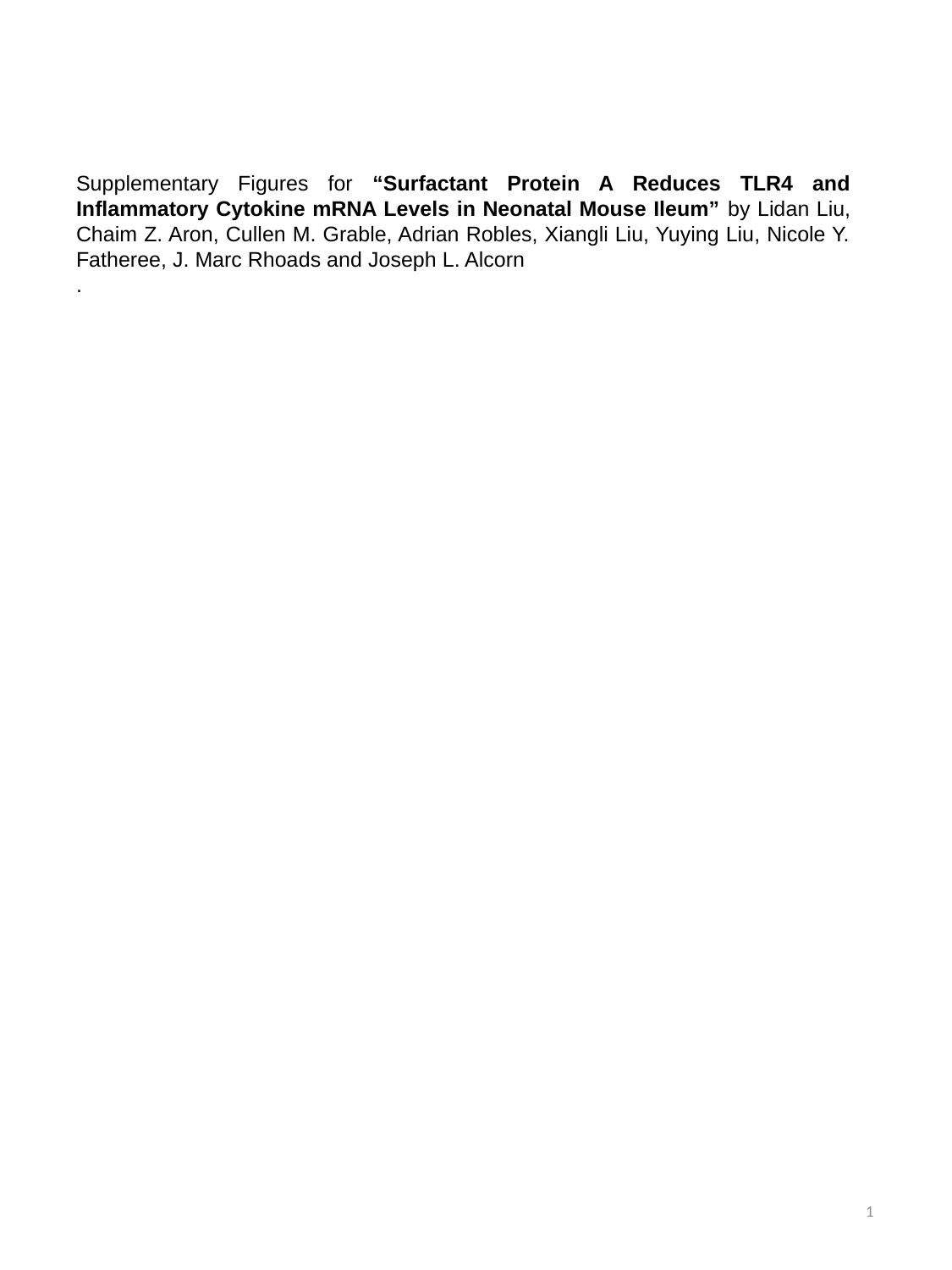

Supplementary Figures for “Surfactant Protein A Reduces TLR4 and Inflammatory Cytokine mRNA Levels in Neonatal Mouse Ileum” by Lidan Liu, Chaim Z. Aron, Cullen M. Grable, Adrian Robles, Xiangli Liu, Yuying Liu, Nicole Y. Fatheree, J. Marc Rhoads and Joseph L. Alcorn
.
1

## Slide 2
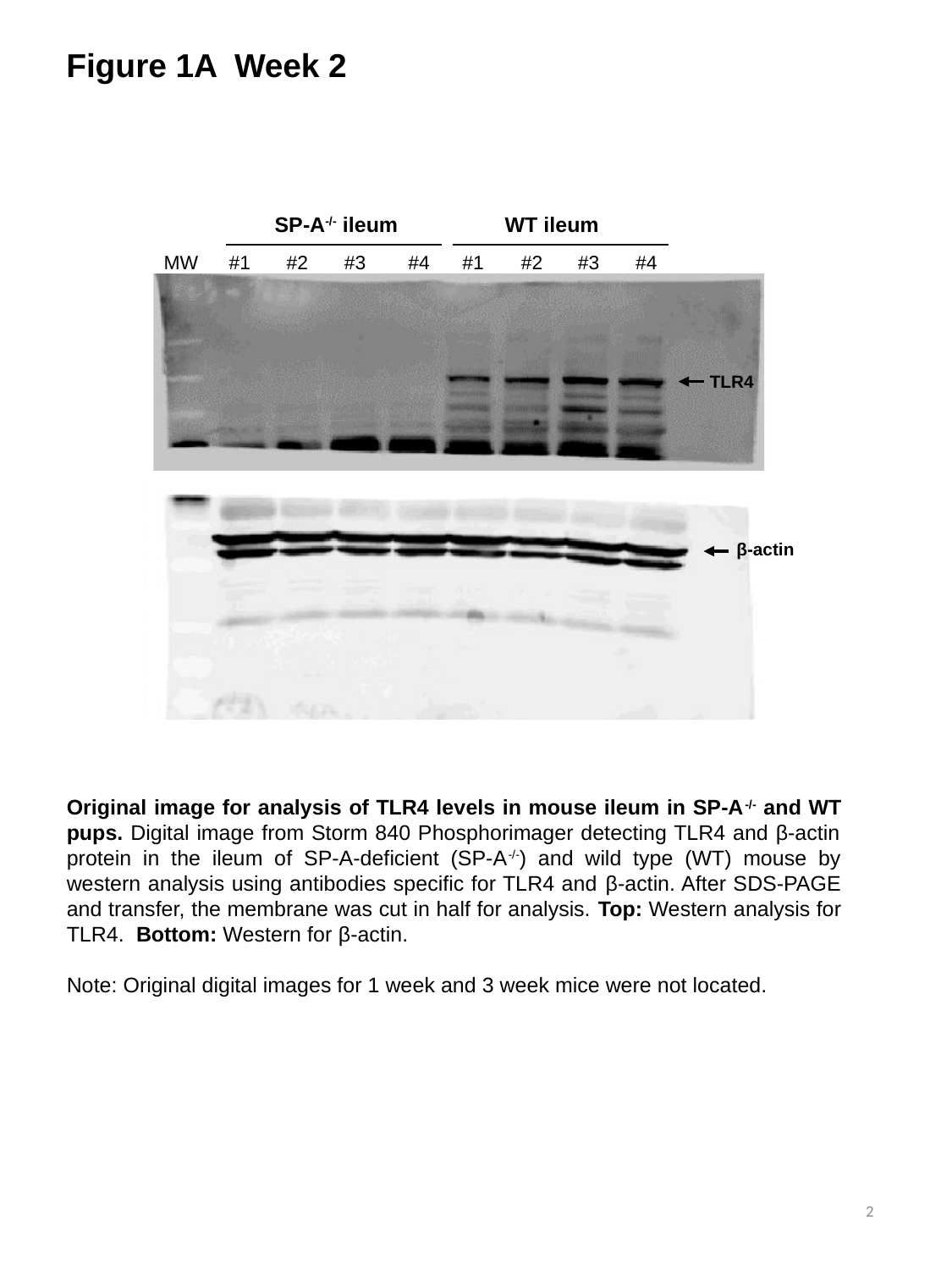

Figure 1A Week 2
SP-A-/- ileum
WT ileum
MW
#1
#2
#3
#4
#1
#2
#3
#4
TLR4
β-actin
Original image for analysis of TLR4 levels in mouse ileum in SP-A-/- and WT pups. Digital image from Storm 840 Phosphorimager detecting TLR4 and β-actin protein in the ileum of SP-A-deficient (SP-A-/-) and wild type (WT) mouse by western analysis using antibodies specific for TLR4 and β-actin. After SDS-PAGE and transfer, the membrane was cut in half for analysis. Top: Western analysis for TLR4. Bottom: Western for β-actin.
Note: Original digital images for 1 week and 3 week mice were not located.
2

## Slide 3
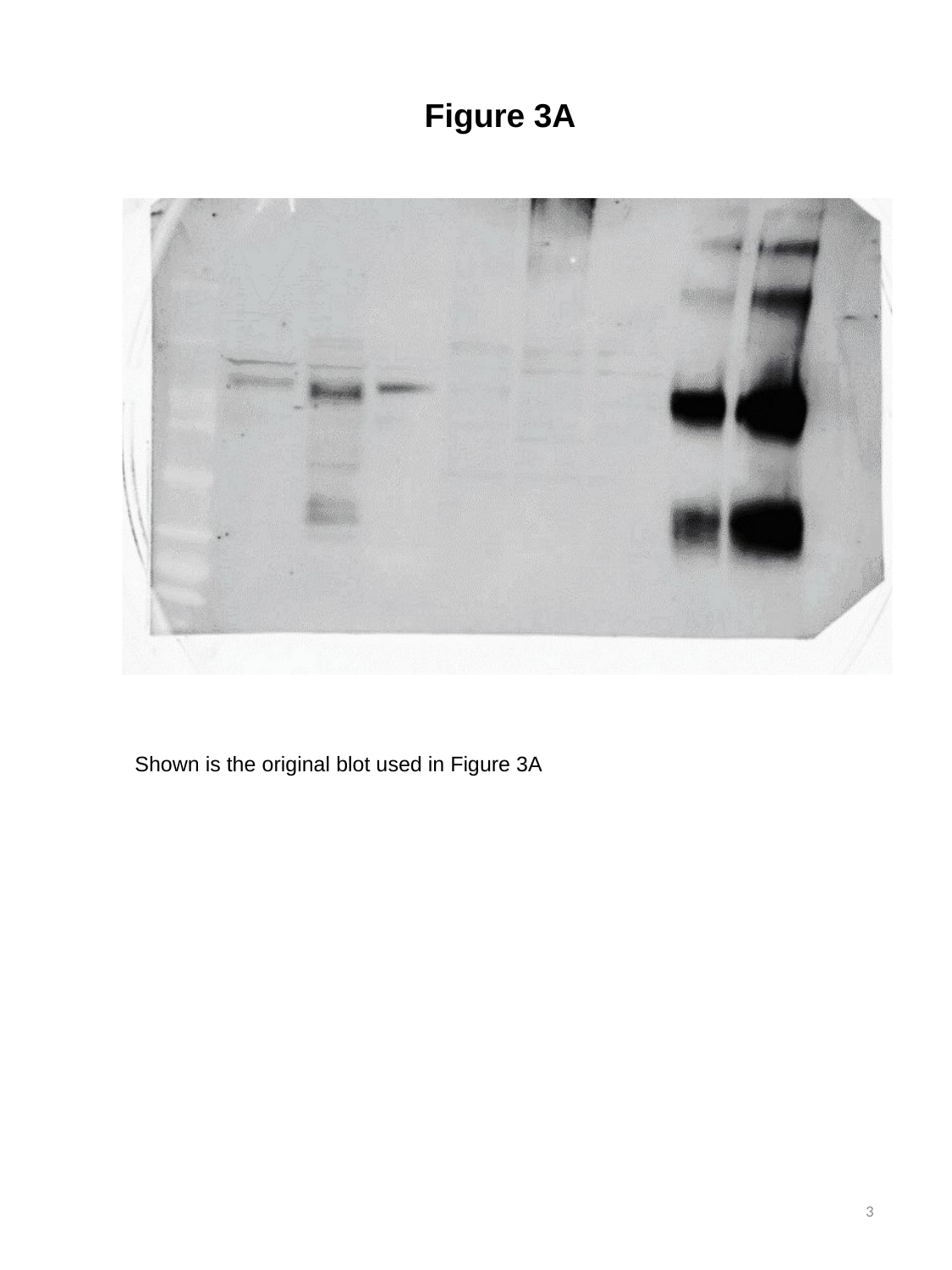

Figure 3A
Shown is the original blot used in Figure 3A
3

## Slide 4
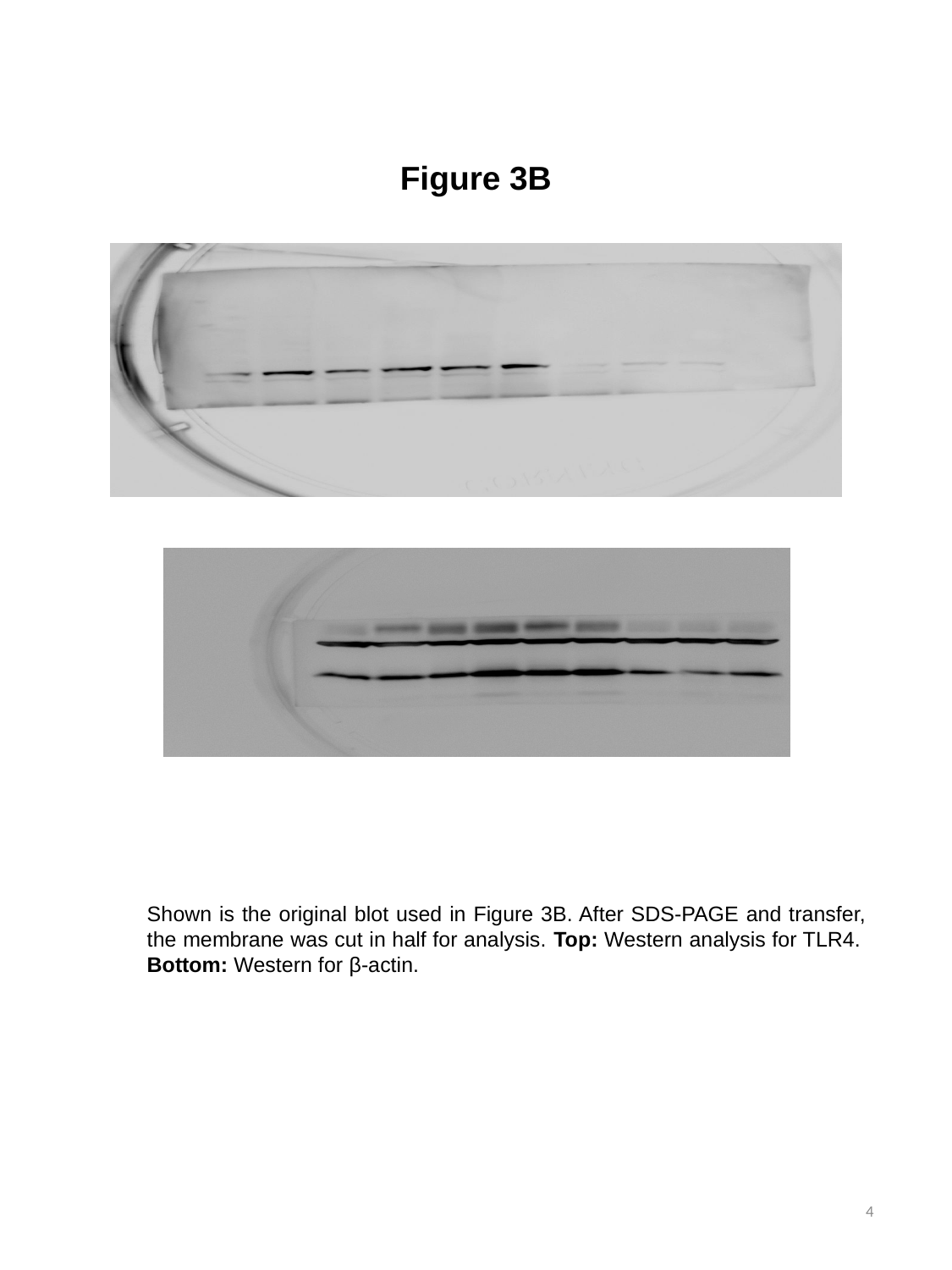

Figure 3B
Shown is the original blot used in Figure 3B. After SDS-PAGE and transfer, the membrane was cut in half for analysis. Top: Western analysis for TLR4. Bottom: Western for β-actin.
4

## Slide 5
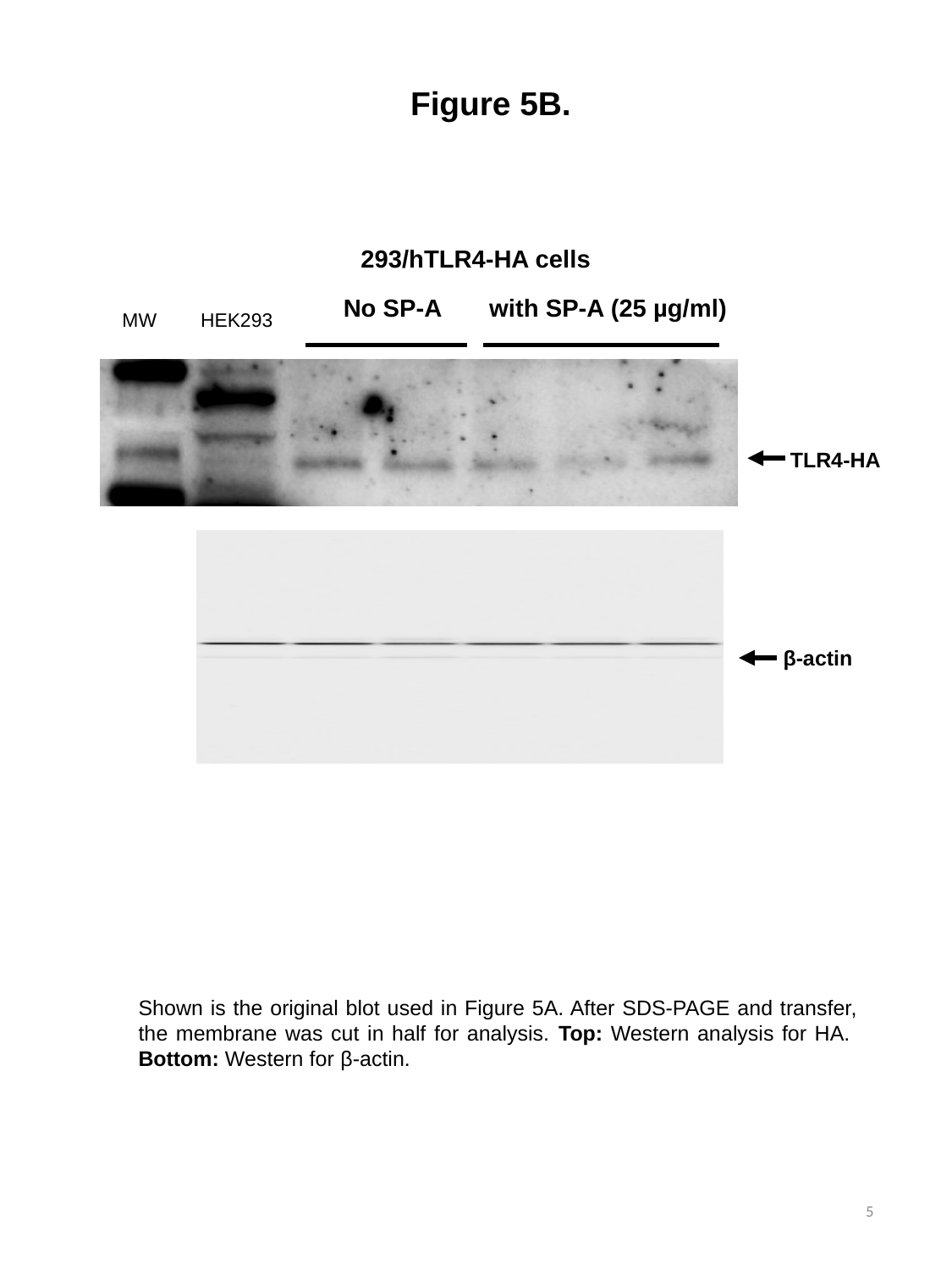

Figure 5B.
293/hTLR4-HA cells
with SP-A (25 µg/ml)
No SP-A
MW
HEK293
TLR4-HA
β-actin
Shown is the original blot used in Figure 5A. After SDS-PAGE and transfer, the membrane was cut in half for analysis. Top: Western analysis for HA. Bottom: Western for β-actin.
5
